# The Effect of the Extra Virgin Olive Oil Minor Phenolic Compound 3′,4′-Dihydroxyphenylglycol in Experimental Diabetic Kidney Disease

**DOI:** 10.3390/nu15020377

**Published:** 2023-01-11

**Authors:** María Dolores Rodriguez-Pérez, Laura Santiago-Corral, Laura Ortega-Hombrados, Cristina Verdugo, María Monsalud Arrebola, Esther Martín-Aurioles, María África Fernández-Prior, Alejandra Bermúdez-Oria, José Pedro De La Cruz, José Antonio González-Correa

**Affiliations:** 1 Departmento de Farmacología, Instituto de Investigación Biomédica (IBIMA), Facultad de Medicina, Universidad de Málaga, 29010 Málaga, Spain; 2 Servicio de Cirugía Plástica, Estética y Reparadora, Hospital Central de la Defensa Gómez Ulla, 28071 Madrid, Spain; 3 UGC Laboratorio Clínico, Hospital de la Axarquía, AGSEMA, 29740 Málaga, Spain; 4 Distrito Sanitario Málaga-Guadalhorce, UGC La Roca, 29009 Málaga, Spain; 5 Consejo Superior de Investigaciones Científicas (CSIC), Instituto de la Grasa, 41013 Seville, Spain

**Keywords:** 3′,4′-dihydroxyphenylglycol, diabetes mellitus, nephropathy, oxidative stress

## Abstract

The aim of this study was to analyze the possible nephroprotective effect of 3’,4’-dihydroxyphenylglycol (DHPG), a polyphenolic compound of extra virgin olive oil (EVOO), on renal lesions in an experimental model of type 1 diabetes. Rats were distributed as follows: healthy normoglycemic rats (NDR), diabetic rats treated with saline (DR), and DR treated with 0.5 mg/kg/day or 1 mg/kg/day of DHPG. DR showed a significantly higher serum and renal oxidative and nitrosative stress profile than NDR, as well as reduced prostacyclin production and renal damage (defined as urinary protein excretion, reduced creatinine clearance, increased glomerular volume, and increased glomerulosclerosis index). DHPG reduced the oxidative and nitrosative stress and increased prostacyclin production (a 59.2% reduction in DR and 34.7–7.8% reduction in DHPG-treated rats), as well as 38–56% reduction in urinary protein excretion and 22–46% reduction in glomerular morphological parameters (after the treatment with 0.5 or 1 mg/kg/day, respectively). Conclusions: DHPG administration to type 1-like diabetic rats exerts a nephroprotective effect probably due to the sum of its antioxidant (Pearson’s coefficient 0.68–0.74), antinitrosative (Pearson’s coefficient 0.83), and prostacyclin production regulator (Pearson’s coefficient 0.75) effects.

## 1. Introduction

Chronic kidney disease is one of the most frequent and serious complications in the evolution of patients with diabetes mellitus [1]. Approximately 30% of patients with type 1 diabetes mellitus and 40% of those with type 2 diabetes mellitus develop some form of chronic kidney disease [2,3]. In general terms, diabetic nephropathy may be defined as a glomerulopathy associated with glomerulosclerosis, with some interstitial and tubular lesions also being observed, although glomerular alterations are the main ones in this process [4].

In the initial stages of diabetic nephropathy, endothelial dysfunction occurs, as well as in the blood vessels of other organs, such as the retina, coronary arteries, etc. [5]. This alteration renders glomerular vessels more sensitive to the effects of vasoconstrictor compounds, such as angiotensin II, which also has a profibrotic effect [6]. The result is an increase in intraglomerular blood pressure and an increase in glomerular filtration characterized by the presence of microalbuminuria [7].

Sustained hyperglycemia is recognized as the main origin of all the alterations that appear in the nephropathic complication of diabetic patients, which initiates the process that induces endothelial dysfunction and activates most of the biochemical mechanisms that lead to glomerular cell damage [3]. One of the biochemical pathways of cellular damage that induce high glucose levels is cellular oxidative stress, a mechanism recognized in almost all tissues of the organism in uncontrolled diabetes mellitus. Oxidative stress was shown to induce glomerular inflammation, which increases glomerular volume; moreover, oxidative stress leads to glomerular function failures, especially an increase in urinary protein excretion and a decrease in glomerular filtration fraction [8,9].

Taking into account the fundamental role of oxidative stress in the onset and development of the renal complications of diabetes, both at the glomerular level and in the rest of the extrarenal blood vessels, the possibility of using antioxidant compounds to prevent these alterations in diabetes mellitus was postulated [10,11,12].

Extra virgin olive oil (EVOO) is an important source of antioxidant substances, mainly polyphenolic compounds, among which hydroxytyrosol stands out for its higher amount compared to the others [13]. It was shown that the administration of hydroxytyrosol to diabetic rats partially prevents the increase in cardiovascular biomarkers of macro- and micro-angiopathic damage, in addition to exerting a neuroprotective and nephroprotective effect [14,15,16,17].

Other polyphenols are present in EVOO, such as 3’,4’-dihydroxyphenylglycol (DHPG) [18], which postulates a possible potentiating effect between these polyphenols, which was demonstrated in brain slices subjected to a hypoxia-reoxygenation experiment in healthy rats, and after oral treatment in type 1 diabetic animals, as well as its effect on cardiovascular biomarkers [19,20,21]. There are no published studies on the possible nephroprotective effect of DHPG.

Therefore, the aim of the study was to analyze the possible effect of 3’,4’-dihydroxyphenylglycol on chronic diabetic kidney disease in an experimental model of type 1 diabetes mellitus. We also aimed to analyze the possible biochemical mechanism that could explain this effect.

## 2. Material and Methods

### 2.1. Study Design

The study was approved by the ethics committee for the Use of Animals of the University of Malaga (Ref. CEUMA31-2018-A) and the Ministry of Agriculture, Livestock, Fisheries, and Sustainable Development, Junta de Andalucía. and Sustainable Development of the Junta de Andalucía] (Ref. 9/07/2019/124).

The experimental animals were 2-month-old Wistar rats. The Spanish regulations for the use and management of experimental animals (EDL 2013/80847, BOE-A-2013-6271) were followed, as well as the recommendations of the guide for the use and care of laboratory animals (NIH publication No. 86-23, revised 1985) and the Spanish Law for the protection of animals.

The experimental groups (*n* = 10 animals per group) were non-diabetic rats (NDR), diabetic rats (DR), and diabetic rats treated with 0.5 or 1 mg/Kg/day of DHPG (in the drinking water). DHPG administration was started 7 days before diabetes induction and continued for 2 months. The dose of DHPG corresponded to the proportion in which it was found with respect to hydroxytyrosol (HT) in EVOO with different polyphenol contents, 1/10 to 1/5 (DHPG/HT) [22,23], and based on previous experiences with HT and DHPG [17,20,21]. For the DHPG dose adjustment, the animals were weighed weekly.

For the induction of experimental diabetes, a single dose of 50 mg/Kg of streptozotocin was administered intraperitoneally. Healthy controls received an equivalent volume of saline. We considered an animal to be diabetic when the blood glucose, measured with a glucose meter, was 200 mg/dL. In order to maintain stable glucose levels (related to increased animal mortality), 4 IU/day of a long-acting insulin analogue was administered (Levemir^®^, Novo Nordisk A/S, Bagsværd, Denmark).

For the animal sacrifice, the animals were anesthetized with intraperitoneal sodium pentobarbital (40 mg/kg weight) and were later decapitated after exsanguination.

### 2.2. Materials

Colorimetric kits and enzyme immunoassay kits were used. Colorimetric kits: thiobarbituric acid reactive substances, total antioxidant capacity (Cell Biolabs Inc., Bionova Científica S.L., Madrid, Spain), and glutathione (Abcam, Cambridge, UK). Enzyme immunoassay kits: 3-nitrotyrosine, 8-iso-prostaglandin F2α (8-isoprostane), 8-hydroxy-2-deoxyguanosine (Cell Biolabs Inc., Bionova Científica S.L., Madrid, Spain), 11-dehydro-thromboxane B2, and 6-keto-prostaglandin F1α (Cayman Chemical Co., Ann Arbor, MI, USA). Sigma Chemical Corp. (St. Louis, MO, USA) provided the rest of the necessary reagents.

3’,4’-dihydroxyphenylglycol (DHPG), with a purity of 95%, was supplied by Fernández-Bolaños J (Instituto de la Grasa, CSIC, Spain). It was obtained from a by-product of olive oil following a two-phase extraction process in oil mills and a subsequent purification process, according to the patent of the same researcher (WO2010070168A1).

### 2.3. Analytical Techniques

#### 2.3.1. Samples

Serum was obtained from non-anticoagulated blood after the centrifugation and separation of the aliquots, which were frozen at −80 °C. For the renal tissue, the right kidney was processed for subsequent histological analysis. The left kidney was processed to determine the biochemical variables; first, the kidney cortex tissue was homogenized in 50 mM phosphate-buffered saline, pH 7.0 (1/15 *w*/*v*), and was later centrifuged at 13,000× *g* for 15 min at 4 °C. The supernatant isolate was aliquoted and frozen at −80 °C. Urine was collected for 24 h in metabolic cages (Tecniplast S.p.A., Buguggiate, Italy). After centrifuging the samples at 3500× *g* for 10 min at 4 °C, they were aliquoted and frozen at −80 °C.

#### 2.3.2. Serum and Urinary Biochemistry

The biochemical parameters that constituted the renal profiles and glucose concentrations were analyzed with the Atellica^®^CH autoanalyzer from Siemens Healthineers (Erlangen, Germany). The creatinine clearance (mL/min/kg body weight) was calculated using the formula described by Giribabu [24] as follows: urine creatinine (mg/dL) × urine volume (mL)/serum creatinine (mg/dL) × 1000/body weight (g) × 1/1440.

#### 2.3.3. Oxidative and Nitrosative Stress

As an index of the lipid peroxide concentration in the serum and renal tissue, the determination of thiobarbituric acid reactive substances was measured since malondialdehyde is the main product of this reaction. We analyzed the serum oxidized low-density lipoprotein as an index of the oxidative state caused by free radicals. We determined the urinary 8-isoprostane concentration for the global quantification of oxidative stress [25]. As an index of oxidative stress-induced DNA damage, 8-hydroxy-2-deoxyguanosine was used. The antioxidant defence was measured using the serum and renal glutathione concentrations and total antioxidant capacity. As an index of nitrosative stress (the formation of peroxynitrites), we measured the concentrations of 3-nitrotyrosine in serum and kidney tissue.

#### 2.3.4. Eicosanoids

The global production of thromboxane and prostacyclin was quantified by determining the urinary concentrations of 11-dehydro-thromboxane B2 and 6-keto-prostaglandin F1α, respectively.

#### 2.3.5. Morphometric Analysis

The right kidney of each rat, after fixation in 10% formalin, embedding in paraffin, and obtaining 5 μm sections, was used for staining with hematoxylin and eosin and periodic acid-Schiff (PAS).

The stained sections were morphometrically analyzed with an image analysis system, including a virtual slide microscope (Olympus BX-UCB, with VS-ASW FL software, Hamburg, Germany). The subsequent quantification was performed with the QuPach-0 program (glomeruli analysis) and the FIJI ImageJ program (https://imagej.nih.gov/ij/download.html, accessed on 10 December 2022) for the morphometric parameters. The glomerular volume (GV) was calculated according to Laneet al. [26]: (GA)3/2 × β/d, where GA is the glomerular area, β is a dimensionless shape coefficient (β = 1.0 for perfect spheres), and d is a size distribution coefficient that adjusts for variations in the glomerular size.

To calculate the glomerulosclerosis rate (GMS), sections stained with PAS (50 glomeruli/section) were used. After assessing the area of each glomerulus, the positive PAS surface was quantified via image analysis using the formula: [PAS(+)A (µm^2^)/GA (µm^2^)] × 100 (GMS: percentage of glomerular area with PAS(+) material; PAS(+)A: area occupied with PAS(+) material in a glomerulus; GA: area of a glomerulus).

### 2.4. Statistical Analysis

A univariate analysis was performed; the data in the text, tables, and figures represent the mean ± standard error of the mean (SEM) of 10 animals. All statistical analyses were performed with the statistical package for social sciences v. 25.0 (SPSS Co., Chicago, IL, USA). The bivariate analysis was conducted using an ANOVA with a subsequent Bonferroni transformation to assess the differences between the treatment groups. Student’s *t*-test was used for the unpaired data in order to establish the difference between the variables analyzed in the NDR vs. DR. The biochemical-morphological correlation was retrieved by calculating the Pearson correlation coefficients. Statistical significance was established using a *p*-value less than 0.05.

## 3. Results

Diabetic rats without treatment with DHPG (DR) showed a lower body weight than normoglycemic animals (NDR), and they ingested more daily chow and water (Table 1). The administration of DHPG did not significantly modify any of these parameters (Table 1).

The kidneys from DR treated with saline showed a significantly higher value (percentage of kidney weight with respect to body weight) compared to the healthy animals (0.9 ± 0.06 in DR versus 0.5 ± 0.04 in NDR, *p* < 0.05). DHPG did not significantly modify the kidney’s dry weight of diabetic animals (0.8 ± 0.06 and 0.8 ± 0.05 with 0.5 and 1 mg/kg/day, respectively).

The serum biochemical profile of the control diabetic animals showed significantly elevated levels of glucose (5.2 times higher) and creatinine (2.3 times higher) compared to normoglycemic animals. DHPG reduced serum glucose and creatinine levels (Table 2).

**Figure 1:**
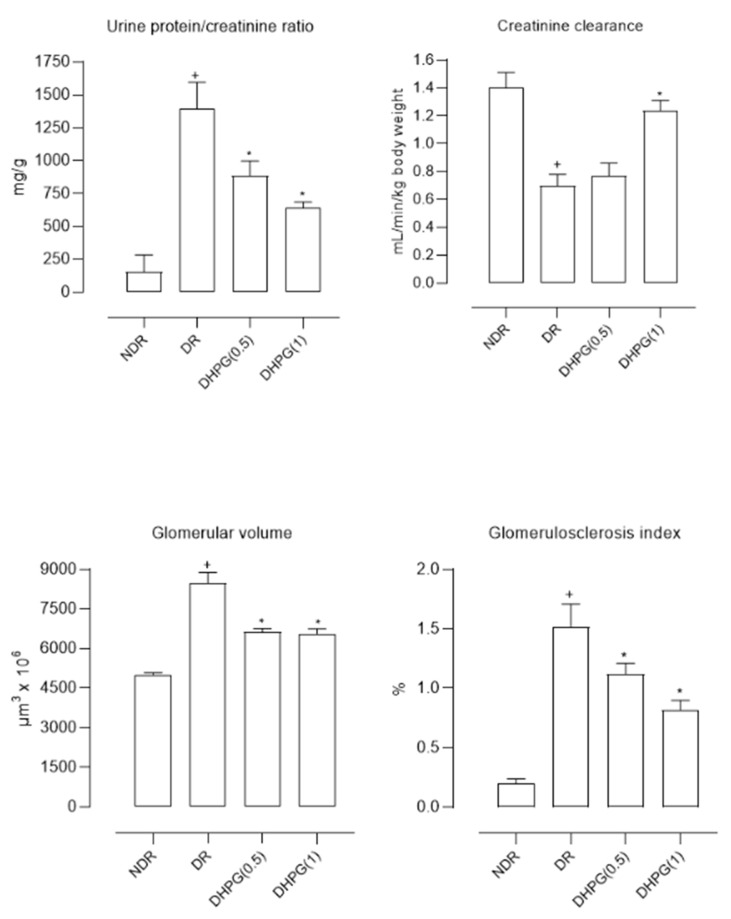
Mean values (mean ± standard deviation) of the urine protein/creatinine ratio, creatinine clearance, glomerular volume, and glomerulosclerosis index in control non-diabetic rats (NDR), control diabetic rats (DR), and DR treated with 3’,4’-dihydroxyphenylglycol (DHPG) 0.5 (DHPG (0.5)) or 1 (DHPG (1)) mg/kg/day p.o. + *p* < 0.05 with respect to NDR. * *p* < 0.05 with respect to DR.

The urine samples from diabetic control animals showed significantly higher glucose concentrations than normoglycemic animals. This glycosuria was not modified by any of the administered DHPG doses. The urinary creatinine excretion was significantly lower in diabetic rats treated with saline than in normoglycemic animals. This excretion was significantly elevated with DHPG 1 mg/kg/day (Table 2). The calculated creatinine clearance was reduced by 53% in diabetic animals treated with saline compared to normoglycemic animals (Figure 1). After two months of administration of DHPG 1 mg/kg/day, this reduction was 14.3% compared to healthy animals. The urinary protein excretion (Table 2 and Figure 1) was 6–7 times higher in diabetic animals, reducing by 38.5% and 64.0% with DHPG 0.5 and 1 mg/kg/day, respectively.

**Figure 2:**
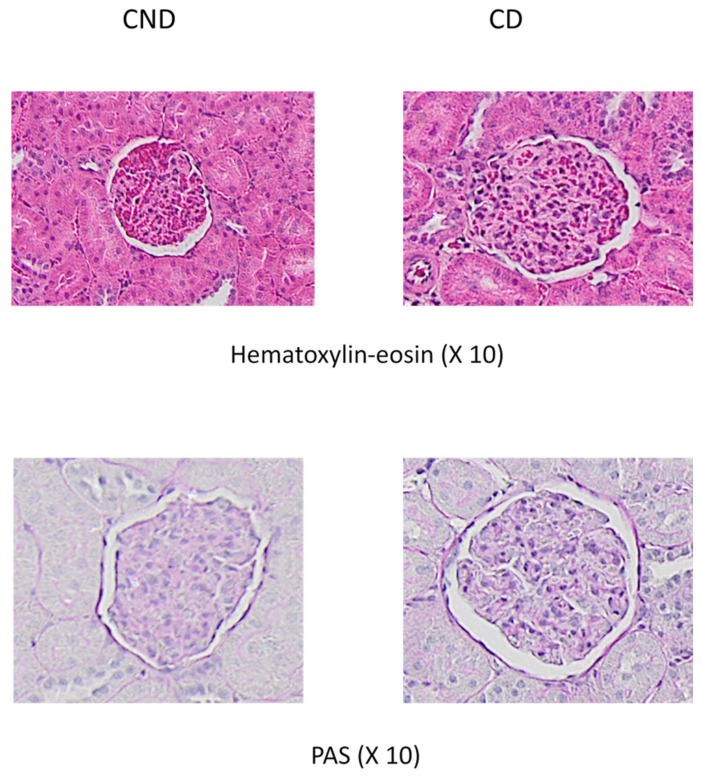
Representative examples of glomerular images from control non-diabetic rats (NDR) and control diabetic rats (DR). Top panels: hematoxylin-eosin staining. Bottom panels: PAS staining.

The glomerular volume was significantly higher in diabetic animals compared to healthy controls (69.4% higher) (Figure 1 and Figure 2), reducing by 22.7% and 22.6% with DHPG 0.5 and 1 mg/kg/day, respectively. This behavior profile was observed with the glomerulosclerosis index, which was significantly higher in diabetic control animals (Figure 1 and Figure 2), decreasing by 26.3% and 46.1% with DHPG 0.5 and 1 mg/kg/day, respectively.

The oxidative stress parameters determined both in serum and in renal tissue were altered in diabetic animals, mainly an increase in those with defined oxidative damage (increases in TBARS, oxLDL, and 8-OHdG) and a decrease in antioxidant defense (GSH and TAC) (Table 3). Likewise, the nitrosative stress parameter (3-nitrotyrosine) increased significantly in control diabetic animals (Table 3). DHPG decreased the values of oxidative and nitrosative damage and slowed down the fall in antioxidant defense.

Prostanoids and 8-isoprostanes syntheses were also altered in diabetic rats treated with saline, mainly higher concentrations of 11-dH-TxB_2_ and 8-isoprostanes in the urine and a reduction in 6-keto-PGF_1α_ production with respect to normoglycemic rats (Table 2). The administration of DHPG decreased the production of 8-isoprostanes and 11-dH-TxB_2_ and stopped the decrease of 6-keto-PGF_1α_.

Table 4 shows the Pearson correlation coefficients between the four renal function parameters (the protein/creatinine ratio in urine, creatinine clearance, glomerular volume, and glomerulosclerosis index) and the biochemical parameters in serum and renal tissue.

## 4. Discussion

This study demonstrates for the first time in the literature that the administration of the extra virgin olive oil minor phenolic compound 3’,4’-dihydroxyphenylglycol partially prevents glomerular kidney damage in an experimental model of type 1 diabetes mellitus. This effect is more clearly shown in the parameters that define renal functionalism, as well as in some variables of oxidative and nitrosative stresses and the production of prostanoids.

Almost 30% of patients diagnosed with type 1 diabetes mellitus suffer from chronic kidney disease in one of its recognized stages [2,3]. Likewise, it is known that glycemic control is the most effective measure to delay this complication and to reduce its intensity and severity if it appears [27]. However, in daily clinical practice, many patients do not strictly control their blood glucose levels, so it is interesting to seek preventive treatments for diabetic nephropathy and, in cases where the metabolic disease is well controlled, to protect the kidney from potential damage during diabetes. Since oxidative stress is a fundamental mechanism in the development of renal lesions in diabetes mellitus [28], it would be justified to study the antioxidant compounds to reduce the influence of this mechanism on kidney damage.

The experimental model used is recognized as representing type 1 diabetes, showing alterations in most of the determined biochemical parameters (Table 2 and Table 3). An increase in oxidative and nitrosative stress, as well as alterations in the production of prostanoids, both in serum and in renal tissue, is accompanied by clear alterations in renal function and morphology (urinary protein excretion, decreased creatinine clearance, increased glomerular volume, and the presence of glomerulosclerosis).

DHPG is an extra virgin olive oil minor phenolic compound with antioxidant and anti-inflammatory effects [18,29,30]. It was demonstrated in smaller amounts than other polyphenols, such as hydroxytyrosol, for which complementary functions were postulated to explain the global antioxidant effect of EVOO. However, DHPG has shown its own effects, both in in vitro and ex vivo experiments, even in a model of type 1 diabetes equal to the one used in this study, although they were quantitatively lower than those shown by HT [19,20,21,29,30]. The results obtained in this study show a qualitatively similar profile to that of HT [17], although with some differences.

First, a decrease in blood glucose levels was quantified, a fact not shown with HT. In this sense, some authors have shown that HT may have a hypoglycemic effect, although in models of type 2 diabetes mellitus [31]. We have not found data about DHPG in the consulted literature, so it is necessary to investigate this effect in other types of experiments. However, sustained hyperglycemia is a fundamental point in the explanation of the evolution of renal lesions in diabetes mellitus, so this effect of DHPG could help in the final nephroprotection.

Second, in the experimental model used, DHPG showed an antioxidant effect on all quantified parameters, although quantitatively less than the antioxidant effect described for HT [17]. It was demonstrated that oxidative stress is a central mechanism in the genesis and development of diabetic nephropathy, explaining some glomerular alterations, such as the excretion of proteins in the urine or the appearance of inflammation, sclerosis, and glomerular edema [28]. Therefore, DHPG, through this antioxidant effect, could also provide a renal protection mechanism. An antioxidant effect for DHPG was demonstrated in other tissues [19,20,21,29,30], although data in kidney tissue have not been published.

Third, DHPG partially reduces the production of thromboxane (a vasoconstrictor prostanoid) and prevents the decreased production of prostacyclin (a vasodilator prostanoid) produced by diabetes mellitus, although these effects were statistically significant at a dose of 1 mg/kg/kg/day (Table 2). An imbalance in the thromboxane/prostacyclin ratio has been classically related to a pro-thrombotic risk [32], so this effect observed for DHPG could also provide another mechanism to explain a possible better state of renal blood flow and decreased in diabetic nephropathy.

Fourth, DHPG modifies the parameters related to renal function since it decreases protein excretion in the urine, increases creatinine clearance, and reduces the increase in glomerular volume and the degree of glomerulosclerosis (Table 2 and Figure 1). The reduction of proteinuria was related to a slowing of the progression of nephropathy; hence this effect of DHPG is important [33]. When performing a statistical analysis that relates these parameters with the biochemical ones (Table 4), the mechanisms for DHPG are significantly related to the findings in the parameters of renal function and morphology, so the final effect of DHPG could be a sum of the modifications performed in these biochemical variables.

An important aspect to consider is the possible function of the effect of DHPG in the demonstrated beneficial effects of EVOO, which is attributed to its polyphenol content. The possibility that DHPG may exert a synergistic effect with HT has been postulated since, in EVOO, both polyphenols are part of the minor components of this oil. In previous studies, it was shown that the cardiovascular and neuroprotective effect of EVOO was not only due to that shown by HT [14,15]. It was shown that DHPG was one of the main polyphenols of EVOO that had an in vitro neuroprotective synergistic effect with HT [19] in a similar proportion to that found in EVOO (1/10-1/20, depending on the type of olives used to obtain EVOO) [22,23].

In our opinion, this study has several limitations, among which we highlight the lack of insight into the molecular nephroprotective mechanisms of DHPG, the need to study these effects in a model of type 2 diabetes, and also we recommend a detailed analysis of the effect of DHPG on blood glucose levels. The necessary studies to respond to these limitations are already underway by our working group.

## 5. Conclusions

The administration of the minor phenolic compound 3’,4’-dihydroxyphenylglycol from extra virgin olive oil to experimental type 1 diabetic rats exerts a nephroprotective effect probably due to the sum of its antioxidant, antinitrosative, and prostacyclin production regulating effects.

## Figures and Tables

**Table 1:** Body weight and daily chow and water intake (mean ± standard deviation) of non-diabetic rats (NDR). Diabetic control rats (DR) and DR were treated with 3′,4′-dihidroxifenilglicol (DHPG) 0.5 or 1 mg/kg/day p.o. (DHPG-0.5. DHPG-1). *N* = 10 rats per group.

Variable	NDR	DR	DHPG-0.5	DHPG-1
Body weight (g)	451 ± 7.1	340 ± 17.1 ^+^	361 ± 17.5	356 ± 14.8
Chow consumption (g/day)	19.11 ± 2.0	28.9 ± 4.5 ^+^	27.6 ± 2.8	23.2 ± 2.4
Drinking water (mL/day)	38.7 ± 14.7	107.8 ± 45.9 ^+^	83.3 ± 8.9	80.8 ± 15.2

^+^ *p* < 0.05 with respect to NDR.

**Table 2:** Serum and urine variables (mean ± standard error of the mean) of non-diabetic rats (NDR). Diabetic control rats (DR) and DR were treated with 3′,4′-dihidroxifenilglicol (DHPG) 0.5 or 1 mg/kg/day p.o. (DHPG-0.5. *N* = 10 rats per group.

Variable	NDR	DR	DHPG-0.5	DHPG-1
Serum				
Blood glucose (mg/dL)	88.2 ± 5.3	461 ± 9.6 ^+^	239 ± 35.7 *	251 ± 35.9 *
Creatinine (mg/dL)	0.3 ± 0.01	0.7 ± 0.03 ^+^	0.5 ± 0.06 *	0.4 ± 0.07 *
Protein (g/dL)	5.6 ± 0.1	5.4 ± 0.2	5.7 ± 0.1	5.6 ± 0.3
Albumin (g/dL)	1.4 ± 0.08	1.4 ± 0.17	1.5 ± 0.06	1.5 ± 0.09
Urine				
Creatinine (mg/dL)	101 ± 7.3	59.4 ± 3.1 ^+^	68.1 ± 9.1	90.0 ± 4.1 *
Proteinuria (mg/L)	13.2 ± 1.1	90.0 ± 9.2 ^+^	55.3 ± 12.3 *	32.4 ± 4.4 *^,a^
Glucosuria (mg/L)	0.0 ± 0.0 ^+^	6483 ± 644	6370 ± 667	5590 ± 493
8-isoprostane (ng/mg creatinine)	6.5 ± 0.5 ^+^	48.1 ± 0.06	4.6 ± 0.7 *	2.8 ± 0.8 *
11-dH-TxB_2_ (ng/mg creatinine)	3.9 ± 1.3 ^+^	9.8 ± 1.0 ^+^	8.4 ± 1.3	6.4 ± 0.5 *
6-keto-PGF_1α_ (pg/mg creatinine)	16.7 ± 0.7 ^+^	6.8 ± 0.9 ^+^	10.9 ± 1.1	15.4 ± 3.4 *

^+^ *p* < 0.05 with respect to NDR; * *p* < 0.05 with respect to DR; ^a^ *p* < 0.05 with respect to DHPG-0.5. 6-keto-PGF_1α_: 6-keto-prostaglandin F_1α_; 8-isoprostane: 8-iso-prostaglandin F_2α_; 11-dH-TxB_2_: 11-dehydro-tromboxane B_2_.

**Table 3:** Serum and kidney variables (mean ± standard error of the mean) of the oxidative and nitrosative stresses of non-diabetic rats (NDR). Diabetic control rats (DR) and DR were treated with 3′,4′-dihidroxifenilglicol (DHPG) 0.5 or 1 mg/kg/day p.o. (DHPG-0.5. DHPG-1). *N* = 10 rats per group.

Variable	NDR	DR	DHPG-0.5	DHPG-1
Serum				
TBARS (nmol/mL)	4.0 ± 0.8	8.7 ± 0.7 ^+^	6.6 ± 0.3 *	6.7 ± 0.5 *
oxLDL (ng/mL)	143 ± 30.8	239 ± 14.0 ^+^	164 ± 22.6 *	174 ± 21.5 *
8-OHdG (ng/mL)	15.8 ± 0.4	25.8 ± 1.5 ^+^	16.1 ± 1.1 *	14.8 ± 1.4 *
GHS (nmol/mL)	124 ± 7.3	89.5 ± 7.0 ^+^	101 ± 3.4 *	108 ± 6.6 *
TAC (U/mL)	17.5 ± 0.5	13.0 ± 0.7 ^+^	12.9 ± 0.6 *	14.9 ± 0.7 *^,a^
3-nitrotyrosine (pg/mL)	14.4 ± 1.0	63.2 ± 3.5 ^+^	53.2 ± 0.8 *	45.5 ± 1.2 *
Kidney				
TBARS (nmol/mg protein)	3.4 ± 0.4	15.6 ± 2.3 ^+^	12.8 ± 1.1 *	12.5 ± 1.0 *
8-OHdG (ng/0.1 g tissue)	6.9 ± 0.4	12.3 ± 0.6 ^+^	10.1 ± 0.3 *	10.5 ± 0.5 *
GHS (µmol/0.1 g tissue)	465 ± 22.3	147 ± 19.7 ^+^	317 ± 22.8 *	399 ± 26.0 *
TAC (U/0.1 g tissue)	85.4 ± 5.3	34.3 ± 7.3 ^+^	63.0 ± 4.1 *	79.7 ± 6.7 *^,a^
3-nitrotyrosine (pg/0.1 g tissue)	20.2 ± 1.3	114 ±10.6 ^+^	88.0 ± 9.1 *	85.8 ± 10.4 *

^+^ *p* < 0.05 with respect to NDR; * *p* < 0.05 with respect to DR; ^a^ *p* < 0.05 with respect to DHPG-0,5. 8-OHdG: 8-hydroxy-2-deoxyguanosine; GSH: reduced glutathione; GSHpx: glutathione peroxidase activity; oxLDL: oxidized low-density lipoprotein; TAC: total antioxidant capacity; TBARS: thiobarbituric acid-reactive substances.

**Table 4:** Statistical Pearson correlations between the glomerular volume (GV), glomerulosclerosis index (GSI), creatinine clearance (CrCl), and the proteinuria/urine creatinine ratio (Prot/Create) with the biochemical variables in serum, kidney, and urine.

Variable	Prot/Creat		CrCl		GV		GSI	
	Pc	*p*	Pc	*p*	Pc	*p*	Pc	*p*
Serum								
TBARS	0.680	0.0001	−0.355	0.064	0.851	0.0001	0.840	0.0001
8-HdG	0.676	0.0001	−0.412	0.030	0.742	0.0001	0.702	0.0001
oxLDL	0.697	0.0001	−0.324	0.093	0.900	0.0001	0.728	0.0001
GSH	−0.736	0.0001	0.500	0.007	−0.865	0.0001	−0.612	0.0001
TAC	−0.752	0.0001	0.549	0.002	−0.817	0.0001	−0.878	0.0001
3-NTy	0.832	0.0001	−0.607	0.0001	0.911	0.0001	0.838	0.0001
Kidney								
TBARS	0.755	0.0001	−0.407	0.032	0.900	0.0001	0.855	0.0001
8-HdG	0.828	0.0001	−0.504	0.006	0.894	0.0001	0.921	0.0001
GSH	−0.820	0.0001	0.577	0.001	−0.876	0.0001	−0.808	0.0001
TAC	−0.861	0.0001	0.665	0.0001	−0.820	0.0001	−0.796	0.0001
3-NTy	0.780	0.0001	−0.474	0.011	0.792	0.0001	0.936	0.0001
Urine								
8-isoprostane	0.743	0.0001	−0.482	0.009	0.797	0.0001	0.647	0.0001
11-dHTxB_2_	0.736	0.0001	−0.506	0.006	0.801	0.0001	0.855	0.0001
6-keto-PGF_1α_	−0.751	0.0001	0.542	0.003	−0.805	0.0001	−0.718	0.0001

Pc: Pearson coefficient. TBARS: thiobarbituric acid reactive substances. 8-OHdG: 8-hydroxy-2-oxyguanosine. oxLDL: oxidized low-density lipoprotein. GSH: reduced glutathione. TAC: total antioxidant capacity. 3-Nty: 3-nitrotyrosine.8-isoprostanes: 8-iso-prostaglandin F_2α_. 11-dHTxB_2_: 11-dehydro-tromboxane B_2_. 6-keto-PGF1α: 6-keto-prostaglandin F_1α_.

## Data Availability

The data presented in this study are available in the article.
